# Hypothermia and coagulation

**DOI:** 10.1186/cc11278

**Published:** 2012-06-07

**Authors:** Kees H Polderman

**Affiliations:** 1 Department of Critical Care Medicine, University of Pittsburgh Medical Center, Pittsburgh, PA, USA

## Introduction

The effects of hypothermia on coagulation may represent a two-edged sword in patients with acute brain injury who are treated with therapeutic cooling. On the one hand inhibition of coagulation can have positive effects, such as improvements in the microcirculation and inhibition of the formation of harmful microthrombi in the brain [1]. On the other hand this could lead to increased bleeding risk and thereby cause harm to patients, especially if they have suffered traumatic injuries or are actively bleeding for other reasons. This manuscript will briefly discuss what is known about the effects of hypothermia on cooling.

## *In vitro *and experimental data

The effects of hypothermia on coagulation have been studied mostly *in vitro*. Very mild hypothermia (down to 35°C) has no effect on any part of the coagulation cascade. Temperatures below 35°C can in some cases (but not in all patients, see below) induce mild platelet dysfunction and sometimes a mild decrease in platelet count. When temperatures drop below 33°C other steps in the coagulation cascade, such as the synthesis and kinetics of clotting enzymes and plasminogen activator inhibitors, can also be affected [2-8]. Recently, Ruzicka and coworkers performed a study in which they precisely measured thromboelastography in healthy subjects at a temperature range starting at 38°C all the way down to 12°C [9]. They reported that decreasing temperatures led to a progressive delay in the initiation of thrombus formation, as well as a decrease in the speed of clot creation and growth. However, significant effects of hypothermia on this parameter began only at 30°C, progressing rapidly below this temperature but reaching statistical significance only at 24°C [9]. These authors also found that once clot formation had been completed, the stability of the clot could no longer be influenced by hypothermia; that is, the clots once formed remained stable regardless of temperature. Of note, there was significant interindividual variability in the response of the coagulation parameters to cooling [9].

Hanke and coworkers [10,11] reported that the anticoagulatory effects of hypothermia were markedly increased if acidosis was present, that the effects of hypothermia could be effectively reversed by administering DDAVP and fibrinogen, but that these drugs worked well only if acidosis was corrected [10].

Finally, a number of animal studies have looked at the effects of hypothermia on hematoma formation in models for intracranial hemorrhage and subdural hematoma [12-16]. These studies have not found any evidence for increased hematoma growth or bleeding risk associated with mild hypothermia; in fact, the opposite effect (decreased hematoma volume and vascular brain edema) was observed in most of these studies [12-16]. Indeed a randomized clinical trial is currently being organized to test the safety and efficacy of cooling in patients with intracranial hemorrhage [17].

## Clinical studies

The clinical effects of mild hypothermia on bleeding appear to be minor, and clinical studies suggest that the risk of severe bleeding associated with mild hypothermia is very low or even absent. None of the large studies in cardiac arrest, stroke, or traumatic brain injury have reported significant increase in bleeding risks associated with therapeutic cooling, although it should be emphasized that actively bleeding patients were excluded from these studies [12].

Preliminary data suggest that hypothermia can even be used safely in combination with thrombolytic therapy. Hemmen and colleagues performed a prospective controlled clinical trial in 58 patients with acute ischemic stroke, 28 of whom were treated with hypothermia (33°C) combined with thrombolytic therapy [18]; they found that the risk of hemorrhagic conversion did not increase in patients treated with both hypothermia and TPA compared to those treated with TPA alone, and in fact the risk of symptomatic ICH trended to be lower in cooled patients [18].

Spiel and colleagues studied the effect of hypothermia induced by cold fluid infusion in cardiac arrest patients; they reported only slightly prolonged clotting time as measured by rotation thrombelastography [19].

Storm and coworkers compared risk and severity of bleeding in cardiac arrest patients treated with mild hypothermia and thrombolysis to matched historical patients treated with thrombolysis only [20]. They found that the incidence of bleeding was not increased by hypothermia, although there was a trend towards more red blood cell units being required to reach target hematocrit in hypothermic patients who *did *develop bleeding complications. As neurological outcomes were significantly better in patients treated with both thrombolytics and hypothermia, even in those who developed bleeding complications, the authors concluded that bleeding risks should not be viewed as a reason to withhold hypothermia treatment [20].

Tuma and colleagues performed a retrospective analysis from a trauma registry database and identified patients who had developed cardiac arrest and had been treated with hypothermia [21]. The number of patients was small but they found no increased complication rate from hypothermia, in particular bleeding, in their patients. This is noteworthy as hypothermia has gained a negative reputation among those treating multi-traumatized patients, and is seen as one of the factors in the 'lethal triad' of shock, acidosis and hypothermia [22]. The discrepancy may be explained by the interplay between acidosis and hypothermia, as explained above; the effects of (deep) hypothermia on coagulation are pronounced, and difficult to reverse, mainly if there is simultaneous acidosis [22]. However, in the absence of acidosis the effects of hypothermia are much less pronounced, and far more easily controllable and reversible. Most patients undergoing elective treatment with mild hypothermia will not have severe acidosis, and therefore effects of hypothermia on coagulation will be minimal.

These clinical data and the physiological effects of cooling should be taken into account when decisions are made on whether or not to use hypothermia in patients who are actively bleeding, or who are at high risk for bleeding. If possible the (potential) source of bleeding should be (surgically) controlled before cooling is initiated. If this is not possible the risks of bleeding should be weighed against the benefits of cooling, and careful consideration should be given to the depth of hypothermia induced in that patient. Because very mild hypothermia (35°C) does not affect coagulation in any patient this temperature can be safely induced even in patients with very high bleeding risk. At temperatures below 35°C some patients may begin to have mild platelet dysfunction (although in most patients this will occur only at lower temperatures). These effects will be significantly magnified in the presence of acidosis.

In my hospital patients who are admitted after cardiac arrest will usually be cooled to 32 or 33°C for a period of 24 hours, followed by slow rewarming over 16 to 24 hours. If such a patient has concomitant severe traumatic injury (for example, blunt abdominal trauma) and/or has injuries that pose a high bleeding risk that cannot easily be surgically controlled (for example, liver laceration), this patient typically would be cooled to 35°C, and cooling would be continued for 48 or 72 hours instead of our current standard of 24 hours. Similarly, if a patient develops severe uncontrollable bleeding during hypothermia therapy (for example, major upper GI bleed with hemodynamic instability) we would consider rewarming this patient to 35°C (or maintaining 33°C and giving platelet transfusion). In mildly elevated bleeding risk we will cool the patient to 33°C and will treat with platelet transfusion and DDAVP if bleeding complications occur (unless there are severe counter-indications such as recent stent placement).

Of note, it is unknown whether decreasing the intensity of cooling while extending the duration of therapy will provide similar benefits as applying treatment according to published guidelines [23]. Therefore, of course those not at high bleeding risk should be cooled to between 32 and 34°C as current guidelines recommend [23].

## Conclusion

Although mild to moderate hypothermia has some effect on the coagulation system the clinical risk of bleeding associated with cooling appears to be very low. This will, however, increase significantly if the patient has moderate-to-severe acidosis. No effects of hypothermia on coagulation occur in any patient as long as temperature is ≥35°C, and patients at very high bleeding risk can safely be cooled to this temperature.
